# *Pseudomonas aeruginosa* enhances anti-PD-1 efficacy in colorectal cancer by activating cytotoxic CD8^+^ T cells

**DOI:** 10.3389/fimmu.2025.1553757

**Published:** 2025-03-21

**Authors:** Lu Chen, Guangcong Ruan, Xuefei Zhao, Ailin Yi, Zhifeng Xiao, Yuting Tian, Yi Cheng, Dongfeng Chen, Yanling Wei

**Affiliations:** Department of Gastroenterology, Chongqing Key Laboratory of Digestive Malignancies, Daping Hospital, Army Medical University (Third Military Medical University), Chongqing, China

**Keywords:** αPD-1, *Pseudomonas aeruginosa*, PA-MSHA, CRC, CD8^+^T cell

## Abstract

**Background:**

Immune checkpoint therapy for colorectal cancer (CRC) has been found to be unsatisfactory for clinical treatment. Fecal microbiota transplantation (FMT) has been shown to remodel the intestinal flora, which may improve the therapeutic effect of αPD-1. Further exploration of key genera that can sensitize cells to αPD-1 for CRC treatment and preliminary exploration of immunological mechanisms may provide effective guidance for the clinical treatment of CRC.

**Methods:**

In this study, 16S rRNA gene sequencing was analyzed in the fecal flora of both responders and no-responders to αPD-1 treatment, and the therapeutic effect was experimentally verified.

**Results:**

*Pseudomonas aeruginosa* was found to be highly abundant in the fecal flora of treated mice, and *Pseudomonas aeruginosa* mannose-sensitive hemagglutinin (PA-MSHA) in combination with αPD-1 was effective in the treatment of CRC through the induction of CD8^+^ T-cell immunological effects.

**Conclusion:**

The clinical drug PA-MSHA can be used in combination with αPD-1 for the treatment of CRC as a potential clinical therapeutic option.

## 1 Introduction

In recent years, colorectal cancer (CRC) has become the third most common form of malignant tumor, following only lung and stomach cancer, and CRC has the second highest mortality rate (1). The preferred method of treatment for CRC is surgical resection combined with radiotherapy and chemotherapy; however, immune checkpoint therapy has recently been incorporated as a supplementary therapy (2). Nevertheless, only approximately 20% of patients with advanced CRC respond to anti-programmed cell death protein 1 antibody (αPD-1) immunotherapy (3, 4). Increasing research revealed that the microbiome plays a pivotal role in tumorigenesis and progression by influencing these processes through inflammatory and immune pathways (1, 5–10). Furthermore, the microbiome has been demonstrated to exerts an essential regulatory influence on the anti-PD-1 response in tumors, including melanoma (11).

A favorable association exists between gut microbiota and immune checkpoint blockade (ICB) efficacy, but the composition of beneficial microbiota may vary across cancer types. *Helicobacter pylori* and *Fusobacterium nucleatum* promote an unfavorable immune response against colon cancer (8, 12, 13). In contrast, *Roseburia intestinalis* and *Lactobacillus johnsonii* play active roles in colon cancer treatment, although their clinical applicability rates are low (14, 15). Fecal microbiota transplantation (FMT) and probiotic transplantation are effective methods for directly improving the gut microbiome. Studies have shown that FMT in combination with αPD-1 is effective against solid tumors, such as melanoma (11, 13, 16). The objective of this study was to investigate the potential of FMT as a biological agent for synergistic αPD-1 therapy in the treatment of CRC. Additionally, this study aimed to identify the most efficacious flora to provide a reference and expand the possibilities for clinical treatment.

In this study, we analyzed fecal samples collected from model mice treated with αPD-1 to assess the flora. We also investigated the ability of the intestinal flora to promote the effects of αPD-1 therapy in patients with CRC through animal model experiments. Our findings suggest that *Pseudomonas* may be a key genus involved in regulating the effects of αPD-1.

## 2 Materials and methods

### 2.1 Mouse models

Male C57BL/6 and BALB/c mice were purchased from Hunan SJA Laboratory Animal Co., Ltd (Changsha, Hunan, China). The mice were housed and reared under specific pathogen-free (SPF) conditions at the Army Specialty Medical Center. All procedures involving animals were performed in accordance with protocols approved by the Chongqing Animal Care and Use Committee.

The mice received antibiotics (ABX) for 7 days. The ABX were a mixture of ampicillin (A9518; Sigma-Aldrich, USA), streptomycin (5711; Sigma-Aldrich, USA), mucomycin (P1004; Sigma-Aldrich, USA), and vancomycin (94747; Sigma-Aldrich). In this study, we developed a mouse model of colon cancer induced by the carcinogens oxidized azomethane (AOM) and dextran sulfate sodium salt (DSS). The mice were anesthetized via an intraperitoneal (i.p.) injection of a mixture of 10 mg/kg AOM (MP Biomedicals). One week after the AOM challenge, the mice received drinking water containing 2.5% DSS for 7 days, followed by a normal diet and drinking water for the next 2 weeks. The DSS challenge was repeated two more times (for a total of 3 cycles of DSS), and the protocol duration was 10 weeks. During third cycle of modeling, mice started to receive FMT treatment daily. Upon completion of the model, the mice were administered an anti-mouse PD-1 monoclonal antibody (BP0146, clone number: RMP1-14, Bio X Cell) or control IgG (BP0089, clone number: 2A-3, Bio X Cell) by intraperitoneal injection (100 μg per mouse, 3 injections in total) every 3 days.

For the subcutaneous mouse model, the MSI-high CRC cell line MC38 (1×10^6^/100 μL of cells per mouse) was inoculated subcutaneously into 8-week-old male C57BL/6 mice, and the CT26 cell line (1×10^6^/100 μL of cells per mouse) was inoculated subcutaneously into 8-week-old male BALB/c mice. Three days after tumor implantation, the mice received daily FMT. One week after implantation, the mice were administered an anti-mouse PD-1 monoclonal antibody or control IgG by intraperitoneal injection (100 μg per mouse, 3 injections total) every 3 days.

For the PA-MSHA (Beijing Wante’er Biological Pharmaceutical Co. Ltd) combined with αPD-1 therapy, approximately 1 × 10^6^ cells were injected subcutaneously into 6–8-week-old male mice. A total of 24 mice were equally divided into four groups: the control group (PBS), αPD-1 (100 μg), PA-MSHA (4 × 10^8^ pcs/mL) and αPD-1(100 μg) plus PA-MSHA (4 × 10^8^ pcs/mL) groups (17). One week after tumor implantation, PA-MSH was injected every five days. Tumor growth was monitored every 2 days by measuring tumor length (L) and width (W). Tumor volume (V) was then calculated via the formula, V = 1/2 × L × W × H. Tumors were collected for pharmacodynamic analysis on day 27. The survival endpoint of tumor-bearing mice was reached when the primary tumor volume exceeded 1000 mm^3^, when the animal demonstrated signs of severe pain and discomfort, or when the animal died because of disease progression.

### 2.2 T-cell isolation and activation

Mouse T cells were isolated from the spleens of naïve mice using the Mouse T-Cell Isolation Kit (19851, Thermo Fisher Scientific) and then cultured in mouse CD3 (14-0032-81, eBioscience), CD28 (14-0281-82, eBioscience) antibody-coated culture flasks with RPMI-1640 medium containing 30 U/mL recombinant mouse IL-2 (51061-MNAE, Sino Biological). T cell differentiation was induced for 48 hours in settings containing different concentrations of PA-MSHA (1 × 10^6^ pcs/ml, 5 × 10^6^ pcs/ml, 1 × 10^7^ pcs/ml, 5 × 10^7^ pcs/ml, or 1 × 10^8^ pcs/ml).

### 2.3 16S rRNA gene sequencing

Fecal samples were collected from both the responder (n=4) and non-responder (n=4) groups of mice. Genomic DNA was extracted from the fecal samples, and its concentration and purity were evaluated using electrophoresis and the NanoDrop 2000 spectrophotometer. PCR amplification was performed on the extracted DNA using Phusion^®^ High-Fidelity PCR Master Mix with GC Buffer (New England Biolabs). Universal 16S rRNA primers targeting the V4 region were utilized for the PCR reaction: 520F (5′-AYTGGGYDTAAAGNG-3′) and 802R (5′-TACNVGGGTATCTAATCC-3′). The resulting PCR products were sequenced on the NovaSeq 6000 platform. Prior to clustering, sequences with quality scores below 20, ambiguous bases, or improper primers were excluded. Chimeric sequences were also identified and removed during the clustering process. High-quality sequences were clustered into operational taxonomic units (OTUs) at a 97% similarity threshold. The similarity in microbial community structure among samples was assessed using principal coordinate analysis (PCoA) based on the Bray-Curtis distance algorithm.

### 2.4 Flow cytometry

Male C57BL/6 mice were sacrificed via cervical dislocation. The tumor tissues were dissected into 1 mm ^3^ pieces and digested with collagenase IV (Worthington) and DNase II (Sigma) for 50 min at 37°C . Digested tumor extracts were filtered through 70 µm cell filters and centrifuged at 1650 rpm for 8 min. Cellular precipitates were collected and resuspended into single-cell suspensions for subsequent antibody labeling.

Antibodies against CD45 (103140), CD69 (104513), CD4 (100509), CD8 (100707), Ly6G (127605), CD11c (117309), CD86 (105007), CD206 (141707) and PD-1 (135229) were purchased from BioLegend. CD3 (45-0036-42), CD19 (12-0193-82) and Granzyme B (396414) were purchased from eBioscience. CD11b (557396), F4/80 (565411), MHC-II (562363), and FoxP3 (562996) antibodies were purchased from BD Biomedicals. The Zombie Aqua™ Fixable Viability Kit was purchased from BioLegend. The tumor cell suspensions were subjected to surface staining with fluorescently labeled antibodies according to the manufacturer’s instructions. Cell viability was assessed using the Zombie Aqua™ Fixable Viability Kit (BioLegend). The cells were subsequently permeabilized using the Transcription Factor Buffer Set (BioLegend) and stained for FoxP3.The cells were analyzed on a CytoFLEX flow cytometer (Beckman Coulter, United States). Flow cytometry data were analyzed using FlowJo software (FlowJo, Ashland, OR, United States).

### 2.5 Statistical analyses

Statistical analysis was performed using GraphPad Prism 9 (GraphPad Software, CA, United States). Mann-Whitney t test was used for comparisons of two groups. Survival data were analyzed via the log-rank (Mantel-Cox) test. The values are expressed as the means ± standard errors of the means (SEM). Two-sided P < 0.05 was considered statistically significant (∗P ≤ 0.05, ∗∗P ≤ 0.01, ∗∗∗P < 0.001). The numbers of animals used in each experiment are indicated in the figure legends.

## 3 Results

### 3.1 Different therapeutic effects of αPD-1 in the treatment of CRC

**Figure 1:**
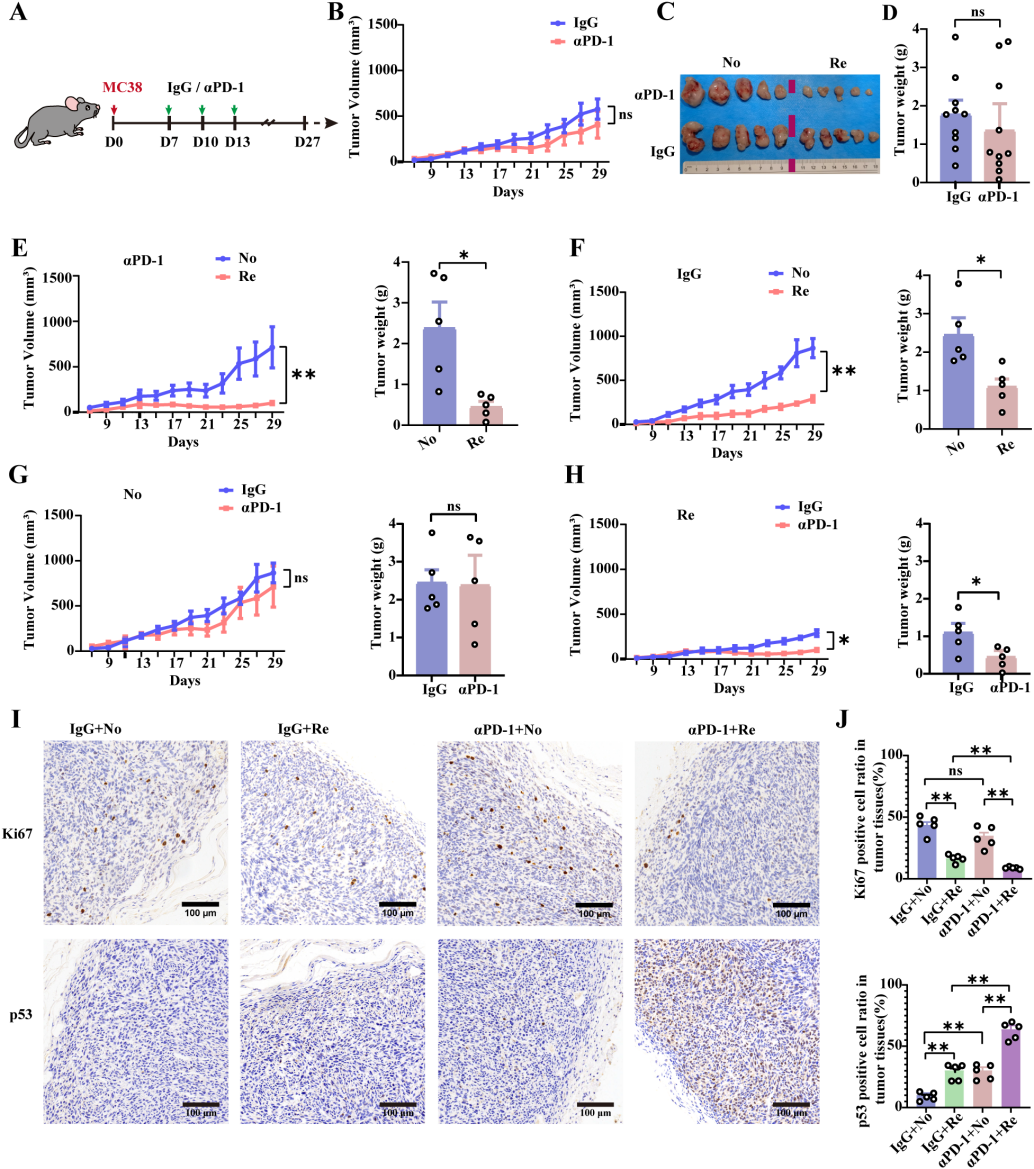
Differential therapeutic effects of αPD-1 treatment in colon cancer model mice. **(A)** Schematic showing the experimental design and schedule. **(B)** Tumor growth curves of αPD-1-treated MC38 hormone-treated mice. On the 27th day after tumor inoculation, tumor tissues were taken for imaging **(C)**, and tumor weight data were obtained **(D)**. n =10 mice per group. Tumor growth curves (left) and tumor loads (right) were counted according to tumor size (RE or NO) for αPD-1 **(E)** or IgG **(F)** treatment effects. Comparison of the therapeutic response of No **(G)** or Re **(H)** mice to αPD-1 therapy based on tumor growth curves (left) as well as tumor loads (right). n =5 mice per group. **(I)** Representative images of Ki-67 as well as p53 IHC staining after αPD-1 treatment (scale bar = 200 μm). **(J)** Statistics of the percentage of Ki67- as well as p53- positive cells. n =5 mice per group. Data represent mean ± SEM., the *P* value was determined by a Mann-Whitney *t* test. ns, not significant; *P < 0.05; **P < 0.01.

CRC is characterized by significantly altered microbiota that regulates the tumor microenvironment. Improving the flora structure is a viable adjuvant option for the treatment of colon cancer. Using the MC38 cell line (18), we modeled aPD-1 therapy for CRC (**Figure 1A**). Tumor volume changes were measured consecutively for 30 days and no significant differences were found between the IgG group and the αPD-1 group (**Figure 1B**). The excised tumor tissue was visually documented, and its mass was recorded (**Figures 1C, D**). The experimental cohorts were subsequently stratified based on the size and mass of tumor tissue, as well as its response to αPD-1. Tumor growth curves and time points were used to divide the mice into two groups, namely, the ‘‘no-responder’’ group and the ‘‘responder’’ group (**Figure 1E**). The tumor weights of no-responsive mice were 4.7 times greater than those of responsive mice. In addition, the tumor growth curves and tumor weights significantly differed between no-responsive mice with rapid tumor growth and responsive mice in the IgG control treatment group, with nonresponsive mice exhibiting 2.18 times greater tumor weights (**Figure 1F**). In addition, tumor tissue growth or weight did not significantly differ between the αPD-1 group and the IgG mAb-treated group (**Figure 1G**). In contrast, tumor tissue growth in responsive mice was inhibited by αPD-1, and the final weight was 0.5 times greater than that in the IgG mAb treatment group (**Figure 1H**).

αPD-1-responsive tumor tissues exhibited a notable decrease in Ki-67 and a notable increase in p53-positive cells (**Figure 1I**). These values were 0.3 times and 1.4 times greater than those of the nonresponsive group, respectively (**Figure 1J**). These data suggest that αPD-1 therapy for CRC is effective, but the observed differences were not significant. This outcome could be attributed to the mice utilized in the study. Certain mice may exhibit characteristics similar to those of αPD-1 treatment-responsive mice, leading to poor growth of subcutaneously implanted colon cancer cells, thereby affecting the T cell-associated immune response in the tumor immune microenvironment.

### 3.2 Fecal flora transplantation from αPD-1-responsive mice in combination with αPD-1 treatment improves survival in a mouse model of CRC

**Figure 2:**
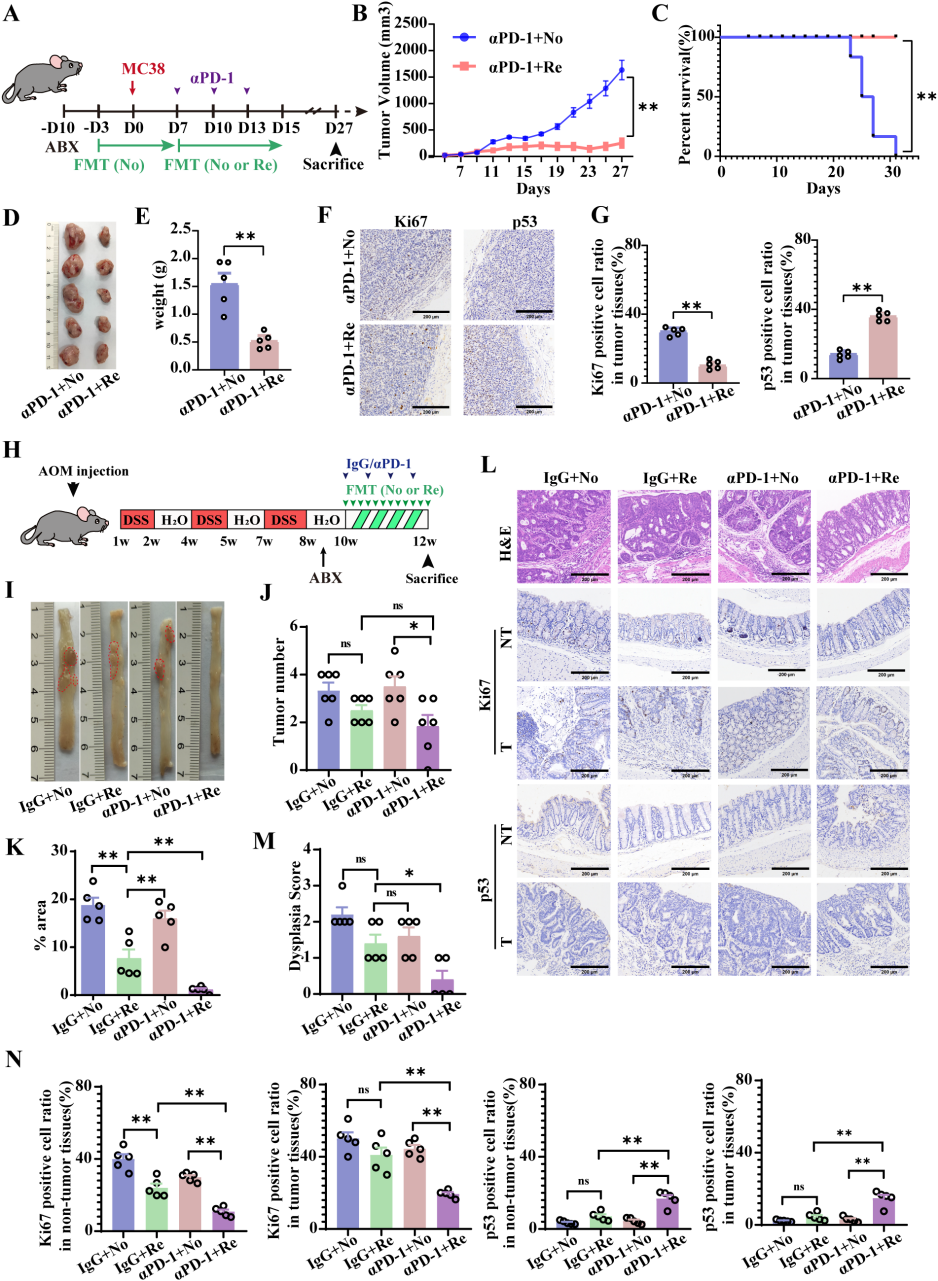
FMT from mice responsive to αPD-1 (Re-FMT) increases the therapeutic potential of αPD-1 in a mouse colon cancer model. **(A)** Schematic showing the experimental design and schedule of the subcutaneous tumor-inoculated mice. Tumor growth curves **(B)** as well as survival curves **(C)** of MC38 hormonal model mice treated with αPD-1 in combination with FMT. On the 27th day after tumor inoculation, tumor tissues were taken for imaging **(D)** as were tumor weight statistics **(E)**. **(F)** Representative imaging of Ki-67and p53 IHC staining after αPD-1 treatment (scale bar = 200 μm). **(G)** Statistics of the percentage of Ki67- and p53-positive cells. n =5 mice per group. **(H)** Schematic showing the experimental design and schedule of primary colon cancer mice. **(I)** Diagram of a representative jointed colon. Number of colon tumors **(J)** and tumor load (total tumor area, mm^2^) **(K)** in model mice. **(L)** Representative H&E staining images and Ki-67 and p53 immunohistochemical (IHC) staining (scale bar = 200 μm). Histopathologic scoring **(M)** of the colon as well as histochemical scoring **(N)**. n =5 mice per group. Data represent mean ± SEM., the *P* value was determined by a Mann-Whitney *t* test. *P < 0.05; **P < 0.01.

Previous studies have shown that gut microbiota plays an important role in supporting immune checkpoint therapies for tumors such as melanoma (5). The role of gut microbiota in promoting tumor progression and improving the efficacy of αPD-1 therapy in CRC needs to be further explored. Sufficient fecal samples were collected from healthy mice before and after tumor inoculation, after which the mice were treated with αPD-1. The mice were then separated into effective and noneffective groups on the basis of their therapeutic response. αPD-1 was combined with fecal microbiota transplantation (FMT) in both effective and noneffective mice to treat MC38-loaded tumor-bearing mice (**Figure 2A**).

FMT intervention in responsive mice (Re-FMT) effectively suppressed the tumor growth and significantly differed from FMT intervention in nonresponsive mice (No-FMT) (**Figure 2B**). Moreover, the survival rate of the mice was significantly enhanced by the combination of Re-FMT and αPD-1 (**Figure 2C**). **Figure 2D** shows an example of tumor tissue from the 27th day after tumor inoculation in the two groups of treated mice. Furthermore, the tumor tissues were weighed, which revealed that the tumor weight of the Re-FMT + αPD-1 combination treatment group was 0.4 times higher than that of the No-FMT + αPD-1 combination treatment group (**Figure 2E**). Moreover, Ki67 was significantly underexpressed, and P53 was significantly overexpressed in the Re-FMT with αPD-1 combination treatment group (**Figures 2F, G**).

A mouse model of primary colon cancer induced by both AOM and DSS validated the promotional role of Re-FMT in αPD-1 tumor therapy (**Figure 2H**). Compared with treatment with No-FMT in combination with αPD-1, Re-FMT in combination with αPD-1 significantly increased the efficiency of targeted therapy (**Figure 2I**) and reduced the number and load of primary colon cancer tumors (**Figures 2J, K**). In addition, mice treated with Re-FMT in combination with αPD-1 also had lower developmental abnormality scores, significantly lower Ki-67 levels in the tumor and non-tumor colon tissues, significantly more P53-positive cells in tumor and non-tumor colon tissues (**Figures 2L-N**), and significantly increased protein expression of the tight junction protein ZO-1 and the mucin MUC2 (**Supplementary Figure 1A, B**). FMT is an important factor in the αPD-1 treatment of CRC and selecting the appropriate donor for FMT will likely support adjuvant αPD-1 colon cancer treatment.

### 3.3 The host intestinal flora modulates the tumor immune microenvironment to influence the αPD-1 response

**Figure 3:**
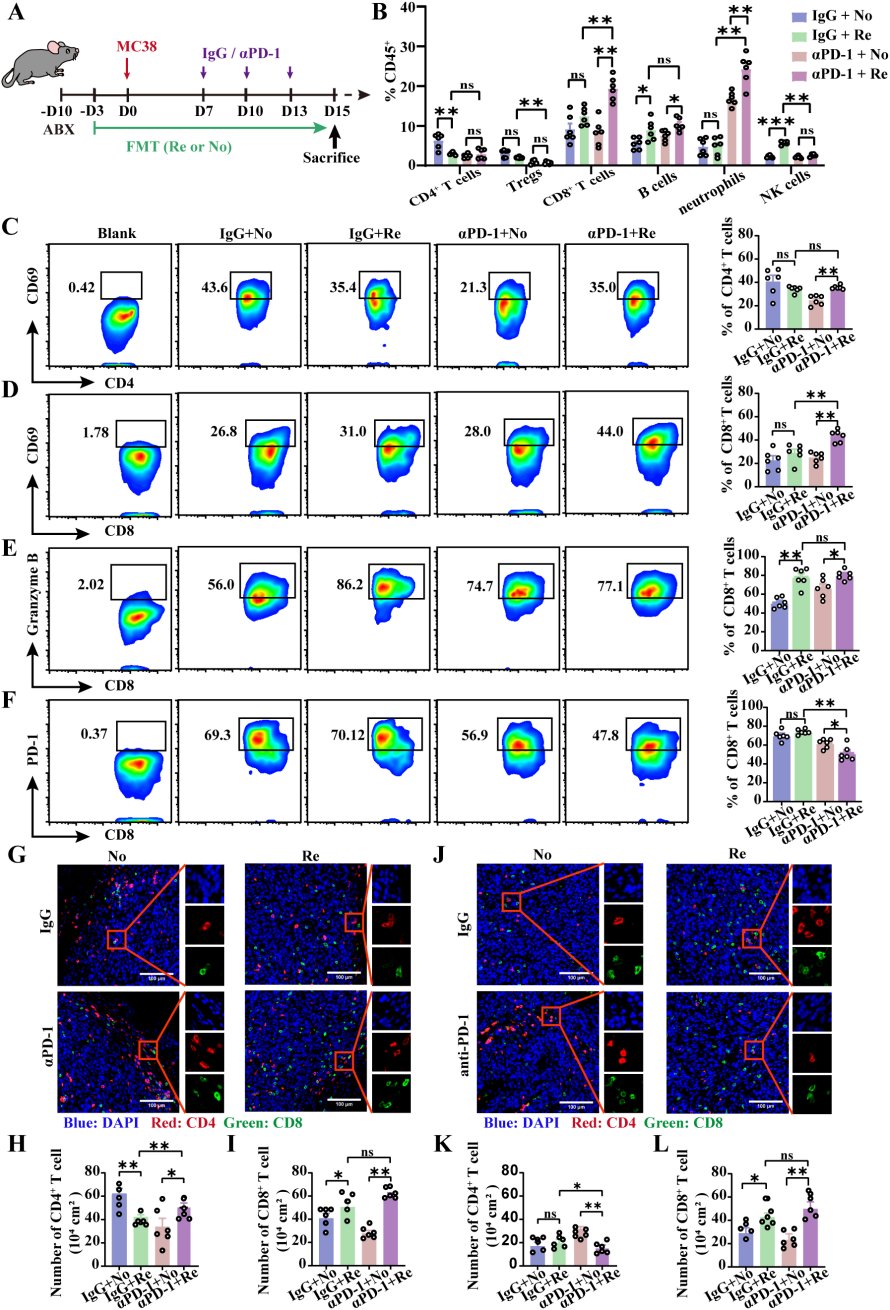
Re-FMT modulates the tumor immune microenvironment in subcutaneous colon cancer. **(A)** Schematic showing the experimental design and schedule of the subcutaneous tumor grafted mice. **(B)** Flow cytometry detection of immune cell recruitment to the tumor microenvironment. **(C)** Representative graphs of CD69 expression in CD4^+^ T cells and positive statistics. Representative graphs of CD69 **(D)**, Granzyme B **(E)**, and PD-1 **(F)** expression in CD8^+^ T cells and positive statistics. **(G)** Representative map of T cell infiltration at the tumor margin. The numbers of CD4^+^ T cells **(H)** and CD8^+^ T cells **(I)** at the tumor margin were counted. **(J)** Representative image of T cell infiltration in the tumor parenchyma. Counts of CD4^+^ T cells **(K)** and CD8^+^ T cells **(L)** in the tumor parenchyma. n =6 mice per group. Data represent mean ± SEM., the *P* value was determined by a Mann-Whitney *t* test. ns, not significant; *P < 0.05; **P < 0.01; ***P < 0.001.

It is imperative to discuss whether FMT could play a key role in promoting tumor progression and improving PD-1 treatment outcomes in patients with colon cancer by modulating the tumor immune microenvironment. The intestinal flora of the mice was modified with ABX and mouse-derived Re-FMT or No-FMT, and the mice were inoculated with MC38 cells and treated with αPD-1 7 days later (**Figure 3A**). Flow cytometry was used to detect immune cell infiltration and the activation of tumor tissue in experimental mice. Compared with αPD-1 and No-FMT treatment, the combination of αPD-1 and Re-FMT significantly inhibited the recruitment of CD4^+^ T cells (especially Tregs) and enhanced the recruitment of CD8^+^ T cells, B cells, and neutrophils in the subcutaneous tumor model (**Figure 3B**). The activated phenotype of CD4^+^ T cells, CD69, was inhibited to some extent by the combination of αPD-1 and Re-FMT (**Figure 3C**). CD69 and Granzyme B were highly expressed in CD8^+^ T cells in the αPD-1 -combined Re-FMT group; compared with the PD1mAb-combined No-FMT, IgG-combined No-FMT and IgG-combined Re-FMT groups, CD69 expression was 1.24-, 1.75- and 1.02-fold higher and Granzyme B expression was 1.95-, 2.22- and 1.81-fold higher, respectively (**Figures 3D, F**). We additionally examined the effects of PD-1 expression on the surface of CD8^+^ T cells induced by αPD-1 and FMT intervention. The experimental results revealed that αPD-1 significantly down-regulated PD-1 expression, whereas Re-FMT intervention further mediated the down-regulation of PD-1 expression on CD8^+^ T cells (**Figure 3F**). Immunofluorescence was used to identify CD4^+^ and CD8^+^ T cell infiltration in the parenchyma and margins of the tumor tissue. Results revealed no significant differences in CD4^+^ T-cell presence at the tumor margins, whereas CD8^+^ T-cell infiltration was considerably higher under αPD-1-responsive conditions (**Figures 3G, I**). Under PD1-responsive conditions, CD4^+^ T-cell infiltration of the tumor parenchyma was reduced, whereas CD8^+^ T-cell infiltration was significantly increased, but there was no significant difference in either case (**Figures 3J–L**).

Regional specificity determines immunological differences between primary colon cancer and subcutaneous tumor models. Therefore, we used a mouse primary colon cancer model to validate the sensitizing effect of Re-FMT on αPD-1 (**Supplementary Figure 2A**). Flow cytometry revealed that compared with αPD-1 combined with No-FMT, αPD-1 combined with Re-FMT inhibited CD4^+^ T cell recruitment to mesenteric lymph nodes, whereas CD8^+^ T cell and B cell recruitment increased (**Supplementary Figure 2B**). In primary colon cancer, the expression of CD69, an indicator of CD4^+^ T-cell activation, was not significantly affected by αPD-1 or Re-FMT (**Supplementary Figure 2C**). However, αPD-1 in combination with Re-FMT significantly up-regulated both activated CD69 and cytotoxic Granzyme B in CD8^+^ T-cell assays (**Supplementary Figures 2D, E**). The expression of PD1 and the effect of αPD-1 were not affected by Re-FMT or No-FMT (**Supplementary Figure 2F**). Consistent with the flow cytometry results, the immunofluorescence results revealed that αPD-1 combined with Re-FMT treatment inhibited CD4^+^ T cell recruitment in colon cancer tissues while promoting CD8^+^ T cell infiltration (**Supplementary Figures 2G-I**). These findings suggest that FMT may assist in tumor treatment by modifying the colon cancer immune microenvironment in response to tumor tolerance. Furthermore, the effectiveness of CD8^+^ T cells against tumor cells was increased by combining FMT therapy with αPD-1.

### 3.4 *Pseudomonas* abundance in the host directly correlates with colon cancer resistance and αPD-1 sensitivity

Numerous experimental results suggest that FMT can be an effective companion for αPD-1 therapy in CRC, improving subcutaneous and primary CRC by remodeling the tumor microenvironment to up-regulate αPD-1 therapeutic response. However, the results also suggest that not all FMTs effectively increase αPD-1 efficacy. The selection of appropriate FMT is a key issue in adjuvant αPD-1 therapy for CRC in the clinic.

**Figure 4:**
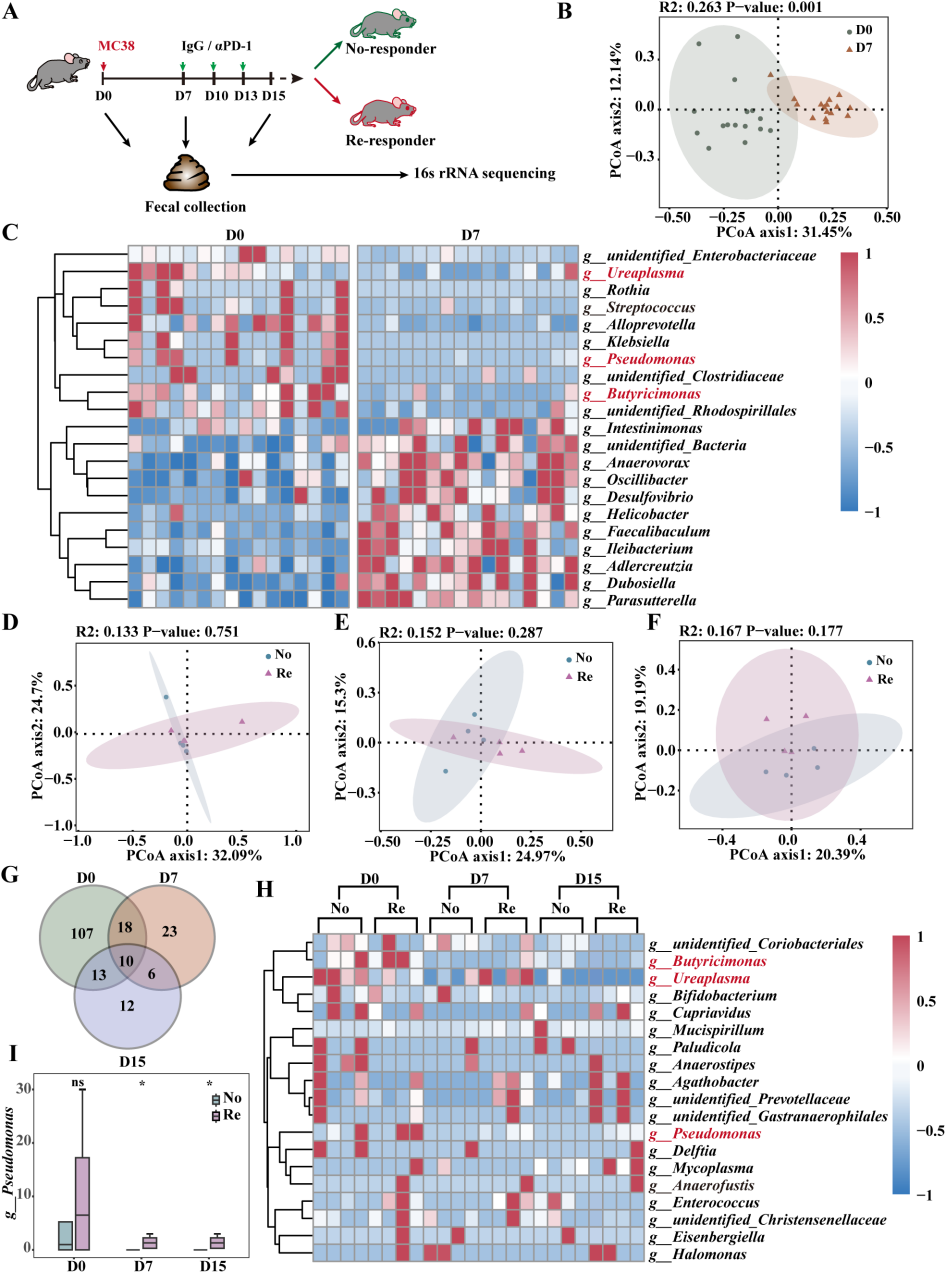
High abundance of *Pseudomonas* in therapeutically effective FMT. **(A)** Schematic diagram showing the process of αPD-1 immunotherapy in the subcutaneous MC38 xenograft tumor model. **(B)** PCoA of 16S rRNA gene sequencing of fecal samples from D0 and D7 mice at the operational taxonomic unit (OTU)level. n =16 mice per group. **(C)** Heatmap demonstrating P<0.05 differential bacterial expression in fecal samples from healthy and tumor-bearing mice. PCoA of 16S rRNA gene sequencing of D0 **(D)**, D7 **(E)** and D15 **(F)** fecal samples from responder mice and no-responder mice at the OTU level. n =4 mice per group. Intersecting genera with more than 2-fold differences in D0, D7 and D15 day means **(G)** and a heatmap of the expression of each genus in the samples **(H)**. The color bar represents the abundance. The relative abundance of *Pseudomonas* **(I)** in the FMT of αPD-1-treated mice was subjected to statistical analysis. n =4 mice per group. Data represent mean ± SEM., the *P* value was determined by a Mann-Whitney *t* test. ns, not significant; *P < 0.05.

Fresh fecal samples were collected from all mice and analyzed using 16S rRNA high-throughput sequencing. The mice were categorized into responders and non-responders based on their therapeutic efficacy. Three distinct time points were established according to tumor load and treatment progression: D0 (baseline, prior to tumor cell inoculation), D7 (load, 7 days post-tumor inoculation), and D15 (treatment, following three rounds of therapy) (**Figure 4A**). Principal coordinate analysis (PCoA) revealed significant differences in β-diversity between D0 and D7 (**Figure 4B**). Furthermore, colony composition analysis at the genus level revealed 33 species of differential genera, with 16 species that exhibited high abundance in healthy mice, including *Alistipes*, *Parabacteroides* and *Pseudomonas* (**Figure 4C**). *Alistipes* and *Parabacteroides* have been demonstrated to increase αPD-1 resistance and influence the prognosis of CRC treatment (19–21). *Pseudomonas* has been shown to possess anti-tumor properties (21), which may be a key factor by which healthy mouse feces can be employed as an adjunct to ICB for CRC.

We compared the differential effects of FMT on αPD-1 and found no significant difference in beta diversity between the Re and No groups at D0, D7, or D15 (**Figure 4D-F**). The intersection of genera with two-fold differences in mean values at each time point was extracted, and a heatmap presented the expression of each genus in the samples (**Figures 4G, H**). The results revealed a high abundance of *Pseudomonas* in the Regroup at all time points (**Figure 4I**, **Supplementary Figures 3A, B**). In addition, the cross-comparison between the two groups (Re and No) for tumor growth differences in the IgG group did not correspond to *Pseudomonas* (**Supplementary Figures 3C-H**). These results suggest that elevating the relative abundance of *Pseudomonas* in the host intestinal microbiota could potentially improve the therapeutic outcomes of αPD-1 treatment for CRC.

### 3.5 *Pseudomonas aeruginosa* mannose-sensitive hemagglutinin combined with αPD-1 effectively treats CRC

*Pseudomonas aeruginosa* (*P. aeruginosa*) *is a common subtype of Pseudomonas* and is usually considered to be associated with clinical infections (22, 23). Recent studies have suggested that *P. aeruginosa* and its secreted cupredoxin azurin are involved in tumor immunity (21). *P. aeruginosa mannose-sensitive-hemagglutinin* (PA-MSHA) is a drug developed based on the anti-tumor properties of *P. aeruginosa* for the treatment of clinical malignancies (17, 24–26). In this study, PA-MSHA was used instead of *P. aeruginosa* to explore its immune effect on αPD-1 in the treatment of CRC.

**Figure 5:**
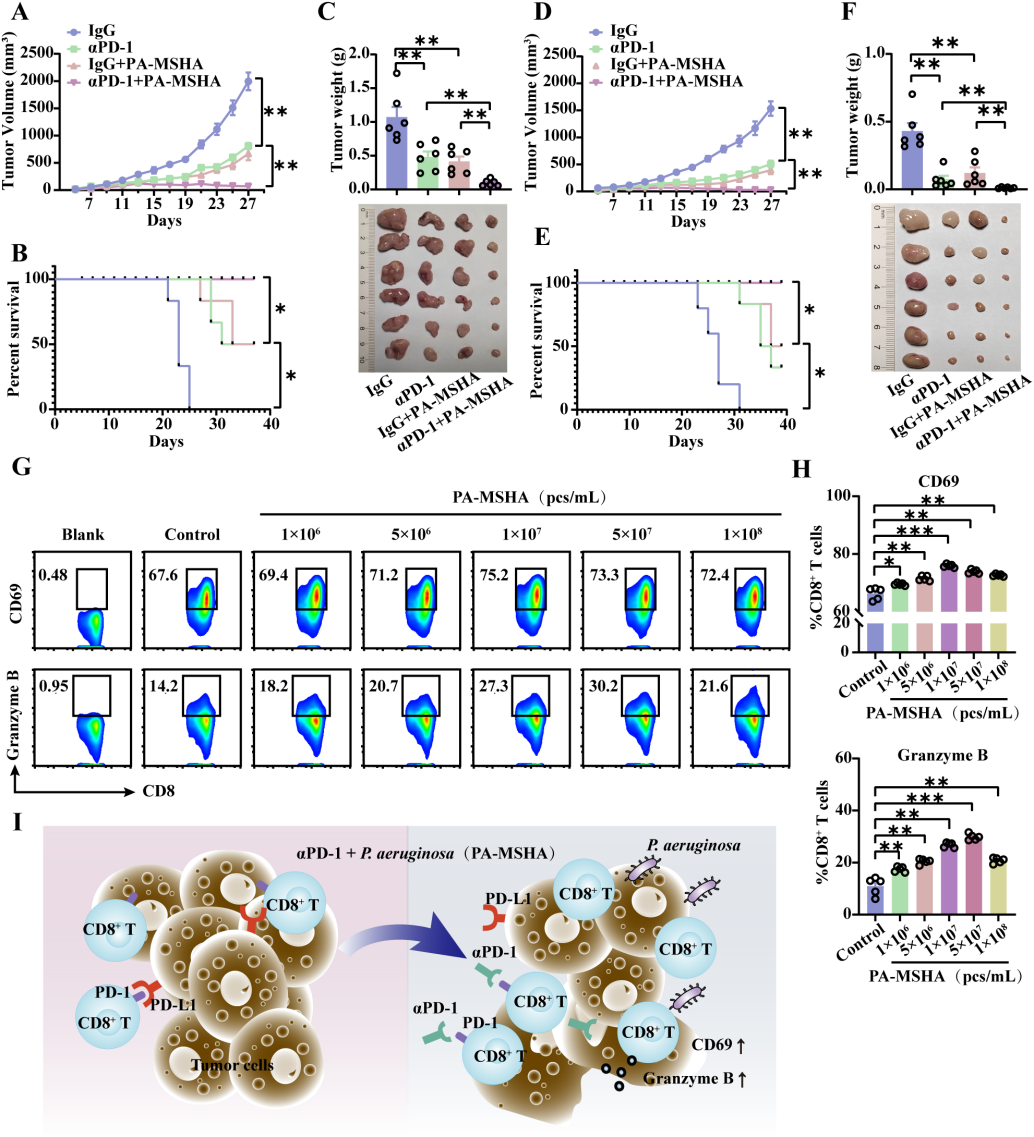
PA-MSHA sensitized cells to αPD-1 for CRC treatment by inducing CD8^+^ T cell activation and cytotoxicity. Tumor growth curves **(A)** and survival curves **(B)** of MC38 hormonal model mice treated with PA-MSHA in combination with αPD-1. **(C)** On day 25, tumors were excised from MC38-loaded mice and subjected to statistical analysis. Tumor growth curves **(D)** and survival curves **(E)** of CT26 hormonal model mice treated with PA-MSHA in combination with αPD-1. **(F)** On day 25, tumors were excised from CT26-loaded mice and subjected to statistical analysis. n =6 mice per group. After PA-MSHA induced CD8^+^ T cells *in vitro* for 48 h, the CD8^+^ T cell activation marker DC69 and the cytotoxicity marker Granzyme B **(G)** were detected by flow cytometry and quantified **(H)**. n=5 per group. **(I)** *Pseudomonas aeruginosa* and its preparation PA-MSHA mediate the therapeutic effects of αPD-1 on CRC by increasing CD8^+^ T cell activation and cytotoxicity. Data represent mean ± SEM., the *P* value was determined by a Mann-Whitney *t* test. *P < 0.05; **P < 0.01; ***P < 0.001.

The therapeutic effect of PA-MSHA in combination with αPD-1 dosing on CRC was evaluated in MC38 and CT26 models. Compared with monotherapy, serial tumor volume measurements demonstrated that αPD-1 in combination with PA-MSHA exhibited a pronounced inhibitory effect on tumor growth (**Figures 5A, D**) and notably increased the survival of MC38- and CT26-loaded mice (**Figures 5B, E**).The tumor tissues from the MC38-loaded mice were photographed on day 25, and the tumor weights were determined, which showed that the tumor masses in the αPD-1 combined with PA-MSHA treatment group were 0.203 and 0.235 times higher than those in the αPD-1 or PA-MSHA alone groups, respectively (**Figure 5C**). Similarly, in the CT26 model, the tumor mass in the αPD-1 combined with PA-MSHA treatment group was 0.15 and 0.09 times higher than those in the αPD-1 or PA-MSHA alone group, respectively (**Figure 5F**). These findings suggest that PA-MSHA significantly enhances the therapeutic effect of αPD-1 on CRC.

Activated CD8^+^ T cells were induced using different concentrations of PA-MSHA, as evidenced by the flow cytometry assay. The expression of the T-cell activation marker CD69 and the cytotoxicity marker Granzyme B was significantly elevated in response to PA-MSHA, with the optimal induction occurring at a concentration of 10^7^ pcs/mL (**Figures 5G, H**). The relevant results further demonstrated that PA-MSHA could enhance the therapeutic effects of αPD-1 in CRC by further mediating CD8^+^ T cell activation as well as Granzyme B expression (**Figure 5I**).

## 4 Discussions

FMT can sensitize αPD-1 to therapeutic effects in a wide range of solid tumors (11, 13–16). Here, we demonstrate the therapeutic effect of FMT-sensitizing PD-1 in CRC and emphasize the significant role of a high abundance of *Pseudomonas* in sensitizing PD-1, thereby advancing our understanding of FMT-assisted PD-1 therapy for CRC. *Pseudomonas* is a genus of gram-negative aerobic or slightly aerobic bacteria belonging to *Proteobacteria*, described in 1894. The most studied species is *P. aeruginosa* (27). In recent years, the worldwide spread of so-called high-risk clones of multidrug-resistant or extensively drug-resistant *P. aeruginosa* has become a public health threat (28, 29). *P. aeruginosa* causes a wide range of acute and chronic infections through the secretion of many cellular immunity-associated virulence factors, and it is multidrug resistant (30–33). The clinical morbidity and mortality rates associated with *P. aeruginosa* infections can reach 40%, with an attributable mortality rate of 13.5%, even when appropriate treatment is administered (34–36).

*P. aeruginosa* traditionally induces apoptosis in cancer cells by secreting the cupredoxin azurin (37–39). Researchers have developed an inactivating agent for tumor therapy based on the cellular immune-inducing properties of *P. aeruginosa*. PA-MSHA is safe and effective as an adjuvant treatment for lung cancer, liver cancer, breast cancer, bladder cancer, and lymphoma (40–45). Zhang et al., based on clinical observations, demonstrated that PA-MSHA-induced cytokine-induced killer (CIK) cells promote the progression of chemotherapy in malignant tumors by mediating CIK cell proliferation and the secretion of pro-inflammatory factors such as IFN-γ (25). In our study, the low abundance of *P. aeruginosa* in the gut microbiota may contribute to the suboptimal efficacy of αPD-1 therapy in CRC, a phenomenon not previously elucidated. Further investigations revealed that the inactivated *P. aeruginosa* preparation, PA-MSHA, enhances the therapeutic effect of αPD-1 by promoting CD8^+^ T cell activity and Granzyme B release. By exploring the role of PA-MSHA in both chemotherapy and immune checkpoint therapy, our findings collectively suggest that PA-MSHA may serve as a promising adjuvant in future clinical cancer treatments. It should be noted that although therapeutic effects were observed in animal models, their clinical translatability necessitates further investigation. Additionally, we have not conducted a thorough evaluation of the potential side effects or consequences associated with the combination therapy.

Recent studies have reported the role of various microorganisms in CRC treatment, including *Roseburia intestinalis* and *Lactococcus lactis*. *Roseburia intestinalis* has been shown to enhance the efficacy of αPD-1 treatment for CRC by inducing functional CD8^+^ T-cell immunity through the metabolite butyrate (14). *Lactococcus lactis* inhibits CRC progression through its production of α-mannosidase (46). Notably, researchers have focused their attention on the role of beneficial bacteria. Indeed, among the FMTs for which αPD-1 treatment was effective, the most significant variability was observed in *P. aeruginosa*, despite its status as a more commonly presumed causative organism in clinical infections.

In conclusion, our findings demonstrate that *P. aeruginosa* sensitizes cells to αPD-1 for the treatment of CRC mediated anti-tumor immune responses by activating cytotoxic CD8^+^ T cells and upregulating Granzyme B expression. Furthermore, PA-MSHA has already received approval from the FDA as a pharmaceutical, suggesting that this novel combination therapy holds promise as an alternative approach to enhancing treatment outcomes, particularly for colorectal cancer patients who have not responded favorably to immunotherapy. This study presents an innovative strategy for integrating bacterial drugs with immune checkpoint inhibitors, offering potential benefits for individuals undergoing cancer immunotherapy.

## Data Availability

The data presented in the study are deposited in the China National GeneBank DataBase (CNGBdb, https://db.cngb.org/) repository, accession number CNP0006126.

## Supplementary material

Supplementary Figure 1 FMT derived from αPD-1-treated mice affects αPD-1 treatment-mediated intestinal barrier function in primary colon cancer. **(A)** Representative MUC2 and ZO-1 immunohistochemical staining images (scale bar = 200 μm). **(B)** MUC2- and ZO-1- positive area statistics. n =5 mice per group. Data represent mean ± SEM., the *P* value was determined by a Mann-Whitney *t* test. ****P* < 0.001; *****P* < 0.0001.

Supplementary Figure 2 Re-FMT modulates the tumor immune microenvironment in primary colon cancer. **(A)** Schematic showing the experimental design and schedule of primary colon cancer mice. **(B)** Flow cytometry detection of immune cell recruitment in mesenteric lymph nodes. **(C)** Representative graphs of CD69 expression in CD4^+^ T cells and positive statistics. Representative graphs of CD69 **(D)**, Granzyme B **(E)**, and PD-1 **(F)** expression in CD8^+^ T cells. **(G)** Representative map of T cell infiltration in the tumor region of colon tissue. Statistics of CD4^+^ T cells **(H)** as well as CD8^+^ T cells **(I)**. n =6 mice per group. Data represent mean ± SEM., the *P* value was determined by a Mann-Whitney *t* test. ns, not significant; * *P* < 0.05; ***P* < 0.01; ****P* < 0.001; *****P* < 0.0001.

Supplementary Figure 3
*Pseudomonas* was significantly different in the αPD-1-treated group but not in the IgG-treated group. The relative abundances of *Butyricimonas* **(A)** and *Ureaplasma* **(B)** in the FMT of αPD-1-treated mice were statistically analyzed. PCoA of 16S rRNA gene sequencing of D0 **(C)**, D7 **(D)**, and D15 **(E)** fecal samples from IgG-responder mice and no-responder mice at the OTU level; n =4 mice per group. Intersecting genera with more than 2-fold differences in D0, D7, and D15 day means **(F)** and heatmaps of the expression of each genus in the samples **(G)**. The color bar represents the abundance, n =4 mice per group. Data represent mean ± SEM., the *P* value was determined by a Mann-Whitney *t* test. ns, not significant; * *P* < 0.05.
