# Supplementary figures and images for: Pseudomonas aeruginosa enhances anti-PD-1 efficacy in colorectal cancer by activating cytotoxic CD8+ T cells
Source: Front Immunol. 2025 Mar 21;16:1553757. doi: 10.3389/fimmu.2025.1553757 (PMC11968734; doi:10.3389/fimmu.2025.1553757)

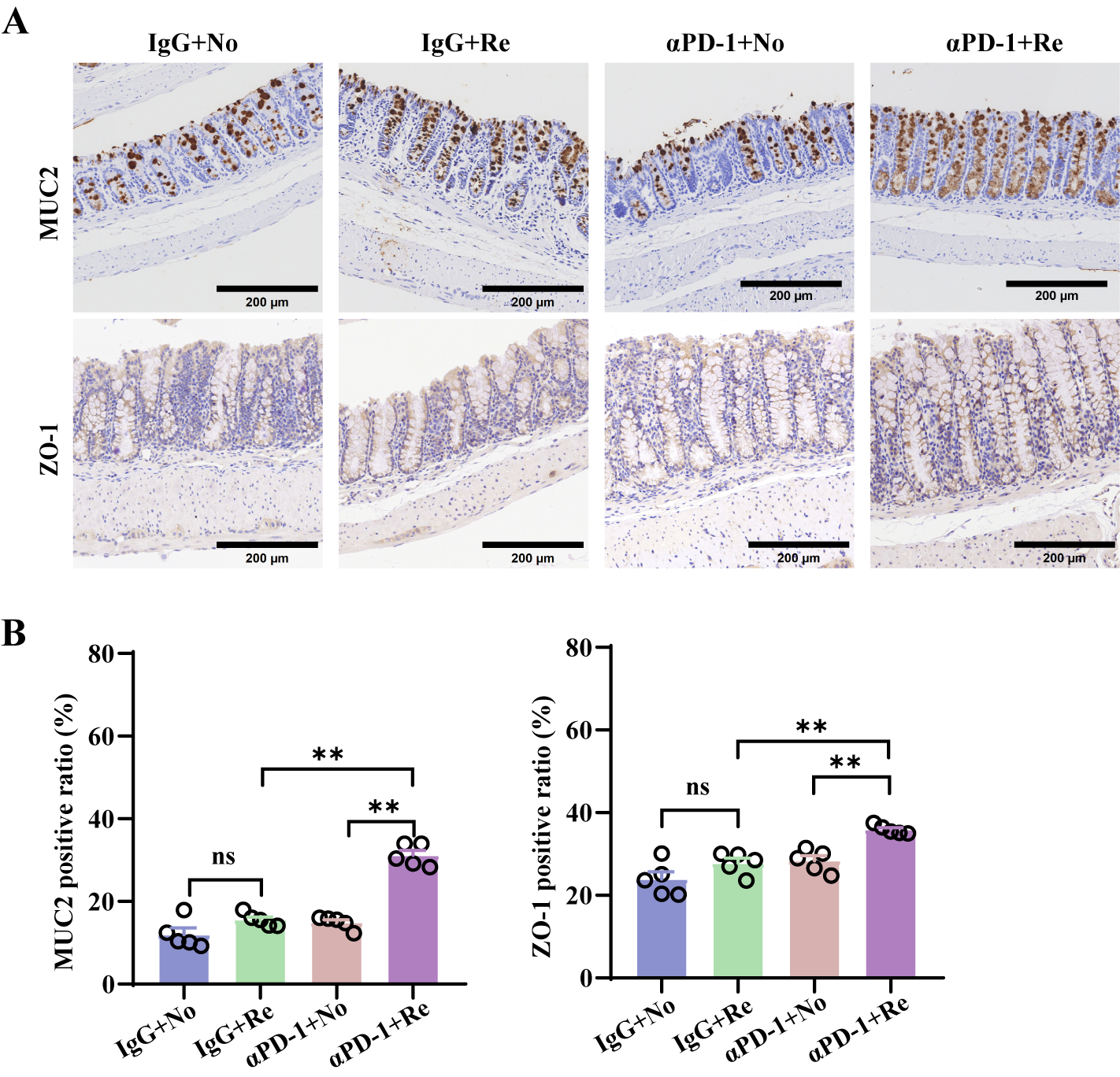

Supplement: Supplementary Figure 1 — FMT derived from αPD-1-treated mice affects αPD-1 treatment-mediated intestinal barrier function in primary colon cancer. (A) Representative MUC2 and ZO-1 immunohistochemical staining images (scale bar = 200 μm). (B) MUC2- and ZO-1- positive area statistics. n =5 mice per group. Data represent mean ± SEM., the P value was determined by a Mann-Whitney t test. ***P < 0.001; ****P < 0.0001. [file Image1.tif]

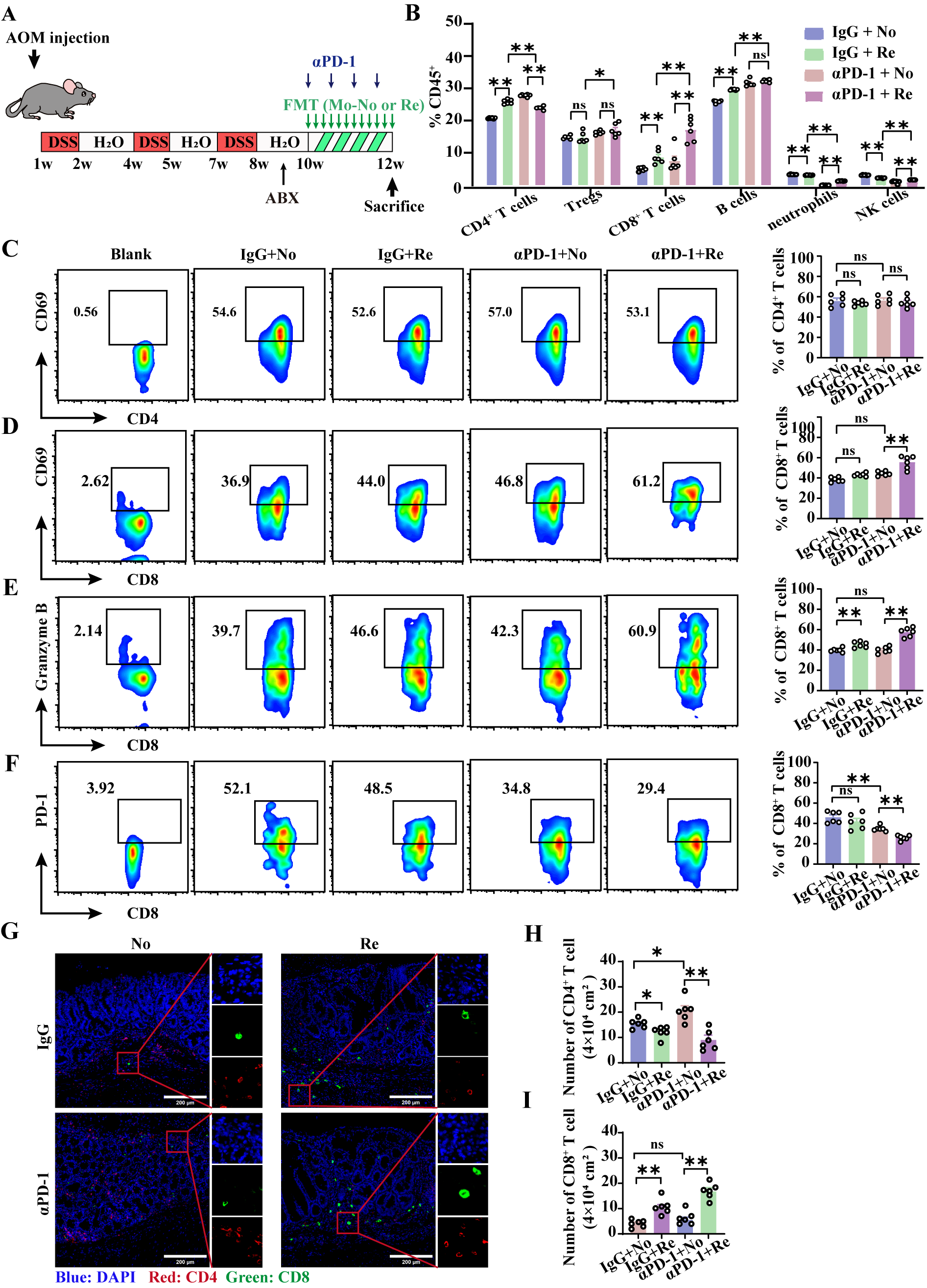

Supplement: Supplementary Figure 2 — Re-FMT modulates the tumor immune microenvironment in primary colon cancer. (A) Schematic showing the experimental design and schedule of primary colon cancer mice. (B) Flow cytometry detection of immune cell recruitment in mesenteric lymph nodes. (C) Representative graphs of CD69 expression in CD4+ T cells and positive statistics. Representative graphs of CD69 (D), Granzyme B (E), and PD-1 (F) expression in CD8+ T cells. (G) Representative map of T cell infiltration in the tumor region of colon tissue. Statistics of CD4+ T cells (H) as well as CD8+ T cells (I). n =6 mice per group. Data represent mean ± SEM., the P value was determined by a Mann-Whitney t test. ns, not significant; * P < 0.05; **P < 0.01; ***P < 0.001; ****P < 0.0001. [file Image2.tif]

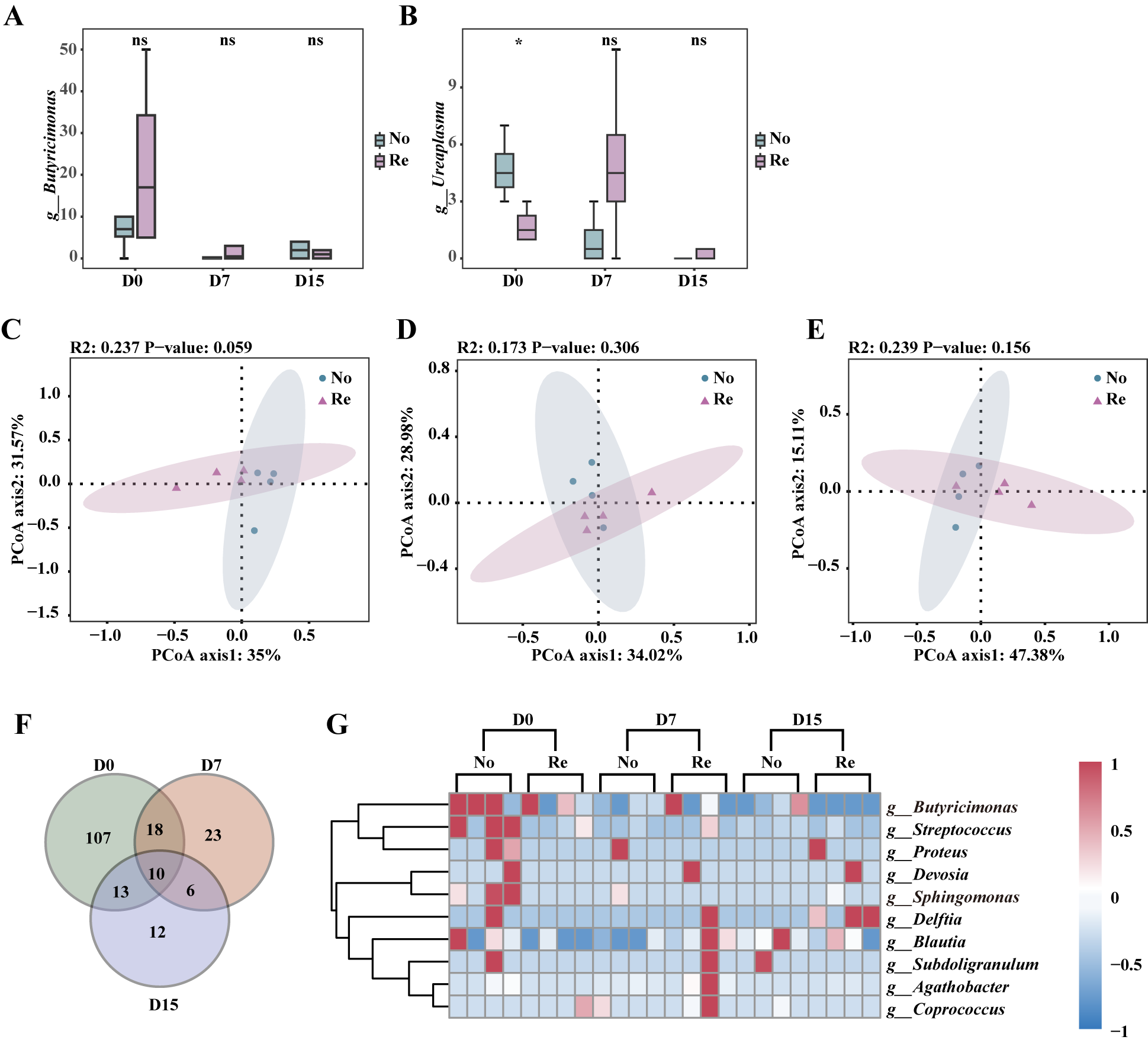

Supplement: Supplementary Figure 3 — Pseudomonas was significantly different in the αPD-1-treated group but not in the IgG-treated group. The relative abundances of Butyricimonas (A) and Ureaplasma (B) in the FMT of αPD-1-treated mice were statistically analyzed. PCoA of 16S rRNA gene sequencing of D0 (C), D7 (D), and D15 (E) fecal samples from IgG-responder mice and no-responder mice at the OTU level; n =4 mice per group. Intersecting genera with more than 2-fold differences in D0, D7, and D15 day means (F) and heatmaps of the expression of each genus in the samples (G). The color bar represents the abundance, n =4 mice per group. Data represent mean ± SEM., the P value was determined by a Mann-Whitney t test. ns, not significant; * P < 0.05. [file Image3.tif]
